# Transition to retirement impact on health and lifestyle habits: analysis from a nationwide Italian cohort

**DOI:** 10.1186/s12889-021-11670-3

**Published:** 2021-09-14

**Authors:** Giacomo Pietro Vigezzi, Giovanni Gaetti, Vincenza Gianfredi, Beatrice Frascella, Leandro Gentile, Angelo d’Errico, David Stuckler, Fulvio Ricceri, Giuseppe Costa, Anna Odone, on behalf of the Italian Working Group on Retirement and Health, Andrea Amerio, Chiara Ardito, Greta Carioli, Giuseppe Costa, Angelo d’Errico, Dario Fontana, Beatrice Frascella, Giovanni Gaetti, Leandro Gentile, Vincenza Gianfredi, Roberto Leombruni, Anna Odone, Fulvio Ricceri, Carlotta Sacerdote, David Stuckler, Giacomo Pietro Vigezzi, Nicolas Zengarini

**Affiliations:** 1 School of Medicine, University Vita-Salute San Raffaele, Milan, Italy; 2 IRCCS Fondazione San Matteo, Pavia, Italy; 3 Department of Epidemiology, ASL TO3, Piedmont Region, Grugliasco, Turin, Italy; 4 Department of Social and Political Sciences, Bocconi University, Milan, Italy; 5 Department of Clinical and Biological Sciences, University of Turin, Turin, Italy; 6 Department of Public Health, Experimental and Forensic Medicine, University of Pavia, via Forlanini, 2, Pavia, Italy

**Keywords:** Ageing, Retirement, Physical activity, Self-rated health, Cohort study, Health behaviour

## Abstract

**Background:**

Retirement is a life-course transition likely to affect, through different mechanisms, behavioural risk factors’ patterns and, ultimately, health outcomes. We assessed the impact of transitioning to retirement on lifestyle habits and perceived health status in a nationwide cohort of Italian adults.

**Methods:**

We analysed data from a large cohort of Italian adults aged 55–70, derived from linking six waves of the Participation, Labour, Unemployment Survey (PLUS), a national survey representative of the Italian workforce population, conducted between 2010 and 2018. We estimated relative-risk ratios (RRR) of transition to retirement and their corresponding 95% confidence intervals (CIs) for selected behavioural risk factors and health outcomes using multivariable logistic regression models. We used propensity score matching (PSM) to account for potential confounders.

**Results:**

We included 5169 subjects in the study population, of which 1653 retired between 2010 and 2018 (exposed, 32%). Transition to retirement was associated with a 36% increased probability of practising sports (RRR 1.36, 95% CI 1.12–1.64). No statistically significant changes were reported for smoking habit (current smoker RRR: 1.18, 95% CI 0.94–1.46) and BMI (overweight/obese RRR: 0.96, 95% CI 0.81–1.15). Overall, retiring was associated with improved self-rated health status (RRR 1.26, 95% CI 1.02–1.58).

**Conclusion:**

Individual data-linkage of multiple waves of the PLUS can offer great insight to inform healthy ageing policies in Italy and Europe. Transition to retirement has an independent effect on perceived health status, physical activity and selected behavioural risk factors. It should be identified as a target moment for preventive interventions, with particular reference to primary prevention so as to promote health and wellbeing in older ages.

**Supplementary Information:**

The online version contains supplementary material available at 10.1186/s12889-021-11670-3.

## Introduction

The world population is rapidly ageing as a result of increasing life expectancy and low fertility, with a faster pace in high-income countries and massive societal impact. It is estimated that by 2050, older people (> 60 years) will account for more than one-fourth of the population in all continents apart from Africa, with peaks at 35% in Europe [1].

As pension reforms across the world attempt to adapt to the ongoing demographic transition and research aims to evaluate their impact on health and welfare, a key fact is that people live long years after retirement compared to the past. Retirement itself is a life-course transition likely to affect behavioural risk factors’ patterns and ultimately health after retirement. Previous research has explored how the transition to retirement modifies selected lifestyle habits, including social networks, smoking [2], alcohol consumption [2], dietary patterns and physical activity [3–5], as well as physical and mental health parameters [6–8]; nonetheless, the available evidence is not conclusive [9, 10]. Indeed, the mechanisms, pull and push factors, of the association between retirement, health and their determinants are complex, while individual, work-related and contextual elements could act as mediators or moderators [11, 12]. It is well known that health and retirement are bi-directionally linked [13]; on one side, retirement (i.e., retirement age and type of retirement) is influenced by health, and, on the other hand, retirement might differentially impact health, depending on different sociodemographic, socioeconomic and psychological factors [10]. After retirement, health and lifestyle may change due to loss of daily routines, physical and mental activity, social interactions and reduction in income. At the same time, moving out from demanding or stressful jobs and having more free time can be beneficial for psychological wellbeing. A negative balance between healthy and unhealthy behavioural patterns associated with retirement might add to the burden of late-life chronic conditions, with ultimate consequences on disability, mortality [14] and high direct and indirect costs.

Retirement is a turning point in people’s life, making the passage into the long last stages of adult life. It cannot be seen as a mere single event, but rather a critical status transition with risks of both positive and negative effects on health, and thus also as a window of opportunity to intervene enabling and supporting healthy behaviours and, more in general, health promotion with preventive purposes [15].

With the general aim of contributing to fill the gaps in knowledge on the changes in health and lifestyles when transitioning to retirement, we investigated the impact of retirement on behavioural risk factors and perceived health in a large cohort of Italian adults.

## Methods

We analysed and critically interpreted data from a large cohort of Italian adults aged 55–70, derived from linking six waves of a national survey representative of the Italian workforce population, conducted between 2010 and 2018. The current study is part of a broader multidisciplinary project on healthy ageing research, the “Pension reforms and spatial-temporal patterns in healthy ageing: quasi-natural experimental analysis of linked health and pension data in comparative Italian and European perspective” (Pe_hA) project, funded by a competitive grant from the Fondazione Cariplo programme on Aging and Social Research [16].

### Data sources

We used data from the Participation, Labour, Unemployment Survey (PLUS) conducted by the Italian National Institute for Public Politics Analysis (INAPP) and included in the Italian National Statistical Program.

PLUS is a national-level survey conducted periodically since 2005 to investigate different aspects of the Italian labour force, focusing on selected subgroups, including workers aged 50 years or more. PLUS waves recruited each year through stratified random sampling from 34,000 to 55,000 subjects, who gave consent to participate and were administered Computer-Assisted Telephonic Interviews (CATI). PLUS includes a classic panel design, as from the second wave in 2006 a relevant sample of the participants (around 60%) was included in the sample of the subsequent year and reinterviewed two or more times in consecutive waves, with a maximum length of the panel from 2005 to 2018. Details on survey design and sampling are available elsewhere [17].

### Study design and outcomes of interest

We linked individual-level data of subjects aged 55–70 across different PLUS waves to build a large cohort study, identifying “transition to retirement” as our exposure of interest. We distinguished between exposed subjects who retired over the study period (i.e., shifted from “employed” to “retired” at two different time points) and subjects who did not (i.e., remained “employed” at two different time points). We considered two subsequent observation time points for both exposed and unexposed subjects (Time0 and Time1).

We focused on the following primary outcomes, related to perceived health and behavioural risk factors’ distribution at Time1, as compared to Time0: rate of change in self-rated health status and physical functioning (no change/improvement/worsening), Body Mass Index (BMI) (overweight-obesity/underweight-normal), smoking habit (yes/no), and physical activity (yes/no). In detail, health status was derived from the self-reported health assessment of the World Health Survey [18], while the reduction in physical functioning was reported as temporary, permanent reduction or none. BMI was calculated from anthropometric data, and behavioural habits (smoking and physical activity) were assessed by dichotomous questions (yes or no) [19].

### Statistical analysis

The characteristics of the study population were explored through descriptive analysis and reported as proportions by exposure status. Since cohort study participants were not randomly allocated to the exposure, we used propensity score matching (PSM) to account for possible selection biases [20]. PSM was conducted separately for men and women. Propensity score (PS) was estimated matching for the following baseline (Time0) covariates, selected on the basis of evidence from the literature and experts’ consultations: age, area of residence, education, type of job and job satisfaction, perceived health status and physical functioning, rate of overweight, smoking habits and physical activity. Details on the questionnaire’s items and categorisation used are provided in Supplementary Table S1. Thus, based on the PS, the distribution of observed baseline covariates resulted homogeneous between exposed and unexposed subjects. Subjects were matched with a calliper width of 0.2 [21]. We compared baseline characteristics of the study population before and after PSM (prevalence and 95% confidence intervals, CIs). Group comparisons were performed using t-test for continuous variables and chi-square for categorical variables.

We estimated relative-risk ratios (RRR) of transition to retirement and their corresponding 95% CIs for various health outcomes comparing Time1 to Time0, using multinomial logistic regression models.

Statistical analyses were conducted using Stata software version 16.0 (Stata Corporation, College Station, Texas, USA).

## Results

After record linkage across different PLUS waves, 5169 subjects were included in the study population, of which 1653 retired between 2010 and 2018 (exposed, 32%) and 3516 did not retire over the study period (unexposed, 68%). In particular, among subjects who retired, 344 (21%) retired between 2010 and 2011, 532 (32%) retired between 2011 and 2014, 419 (25%) retired between 2014 and 2016, and 367 (22%) between 2016 and 2018.

Table 1 shows study population baseline characteristics, by exposure status, before and after PSM. Prior to PSM, the mean age was 60.37 years in the exposed group and 58.15 years in the unexposed group. There were 976 males (59%) and 677 females (41%) in the exposed group, and 2043 males (58.1%) and 1473 females (41.9%) among the unexposed. In the exposed group, before retirement, 73.7% of subjects (*n* = 1218) were white-collar workers and 26.3% (*n* = 435) blue-collar workers; in the unexposed group 78.2% were white-collar, 21.8% blue-collar (*p* < 0.01). Job satisfaction was mainly medium-high in both groups (around 59%): the exposed group was more frequently highly satisfied, and the unexposed group was more frequently medium-low and low satisfied (*p* = 0.05). Education level was mostly medium in both groups (46% vs 47.2%): the exposed group was less educated, and the unexposed group had higher educational levels (*p* < 0.01). Self-reported health was mostly good-excellent in both groups (65.2% vs 66.3%). Self-reported BMI was overweight-obese in 52.6% in the exposed group and 50.5% in the unexposed group. Smoking habit was reported by around 20% and sports habit by around 30% in both groups. Limitations to physical functioning reduction (partial or total) were reported by around 7% of the sample, both in the exposed and unexposed groups.

**Table 1:** Baseline characteristics of the study population before and after propensity score matching by exposure status

	Before PSM			After PSM		
	Transition to retirement n. (%)	Control n. (%)		Transition to retirement n. (%)	Control n. (%)	
**n.**	1653	3516	*p-value*^*a*^	1635	1635	*p-value*^*a*^
***Age (mean, years)***	60.37	58.15	< 0.01*	60.36	60.39	0.78
***Gender***			0.52			1
*Males*	976 (59.0%)	2043 (58.1%)		968 (59.2%)	968 (59.2%)	
*Females*	677 (41.0%)	1473 (41.9%)		667 (40.8%)	667 (40.8%)	
***Job***			< 0.01*			1
*White collar*	1218 (73.7%)	2751 (78.2%)		1206 (73.8%)	1206 (73.8%)	
*Blue collar*	435 (26.3%)	765 (21.8%)		429 (26.2%)	429 (26.2%)	
***Job satisfaction***^***b***^			0.05			0.71
*High*	338 (20.6%)	619 (17.8%)		336 (20.5%)	315 (19.3%)	
*Medium-high*	982 (59.8%)	2091 (60.2%)		977 (59.8%)	1008 (61.7%)	
*Medium-low*	251 (15.3%)	599 (17.2%)		250 (15.3%)	252 (15.4%)	
*Low*	72 (4.3%)	168 (4.8%)		72 (4.4%)	60 (3.6%)	
***Education level***			< 0.01*			0.47
*High*	475 (28.7%)	1268 (36.1%)		467 (28.6%)	440 (26.9%)	
*Medium*	760 (46.0%)	1661 (47.2%)		757 (46.3%)	802 (49.1%)	
*Low*	418 (25.3%)	587 (16.7%)		411 (25.1%)	393 (24.0%)	
***Area of residence***^***c***^			0.01*			0.63
*North West*	389 (23.5%)	741 (21.1%)		382 (23.4%)	387 (23.7%)	
*North East*	360 (21.8%)	649 (18.4%)		353 (21.6%)	315 (19.3%)	
*Center*	337 (20.4%)	759 (21.6%)		336 (20.5%)	344 (21.0%)	
*South and islands*	567 (34.3%)	1367 (38.9%)		564 (34.5%)	589 (36.0%)	
***Self-reported health status***			0.75			0.78
*Excellent*	238 (14.4%)	493 (14.0%)		234 (14.3%)	212 (13.0%)	
*Good*	839 (50.8%)	1839 (52.3%)		832 (50.9%)	863 (52.8%)	
*Satisfactory*	392 (23.7%)	816 (23.2%)		386 (23.6%)	375 (22.9%)	
*Poor-bad*	184 (11.1%)	368 (10.5%)		183 (11.2%)	185 (11.3%)	
***BMI***			0.17			0.80
*Underweight-normal*	784 (47.4%)	1740 (49.5%)		776 (47.5%)	767 (46.9%)	
*Overweight-obese*	869 (52.6%)	1776 (50.5%)		859 (52.5%)	868 (53.1%)	
***Current smoker***^***b***^			0.10			0.04*
*Yes*	378 (22.9%)	727 (20.9%)		374 (22.9%)	314 (19.2%)	
*No*	1271 (77.1%)	2751 (79.1%)		1261 (77.1%)	1321 (80.8%)	
***Sports habit***^***b***^			0.32			0.36
*Yes*	541 (32.8%)	1092 (31.4%)		530 (32.4%)	499 (30.5%)	
*No*	1110 (67.2%)	2389 (68.6%)		1105 (67.6%)	1136 (69.5%)	
***Physical functioning***			0.29			0.79
*Partial limitation*	50 (3.0%)	121 (3.4%)		49 (3%)	41 (2,5%)	
*Total limitation*	61 (3.7%)	104 (3.0%)		59 (3,6%)	59 (3,6%)	
*No limitation*	1542 (93.3%)	3291 (93.6%)		1527 (93,4%)	1535 (93,9%)	

^a^T-test was used for continuous variables, chi-square for categorical variables

^b^missing data not reported (59 for job satisfaction, 42 for current smoker, 37 for sports habit)

^c^within Italy

*PSM* Propensity score matching, *BMI* Body mass index

**Fig. 1:**
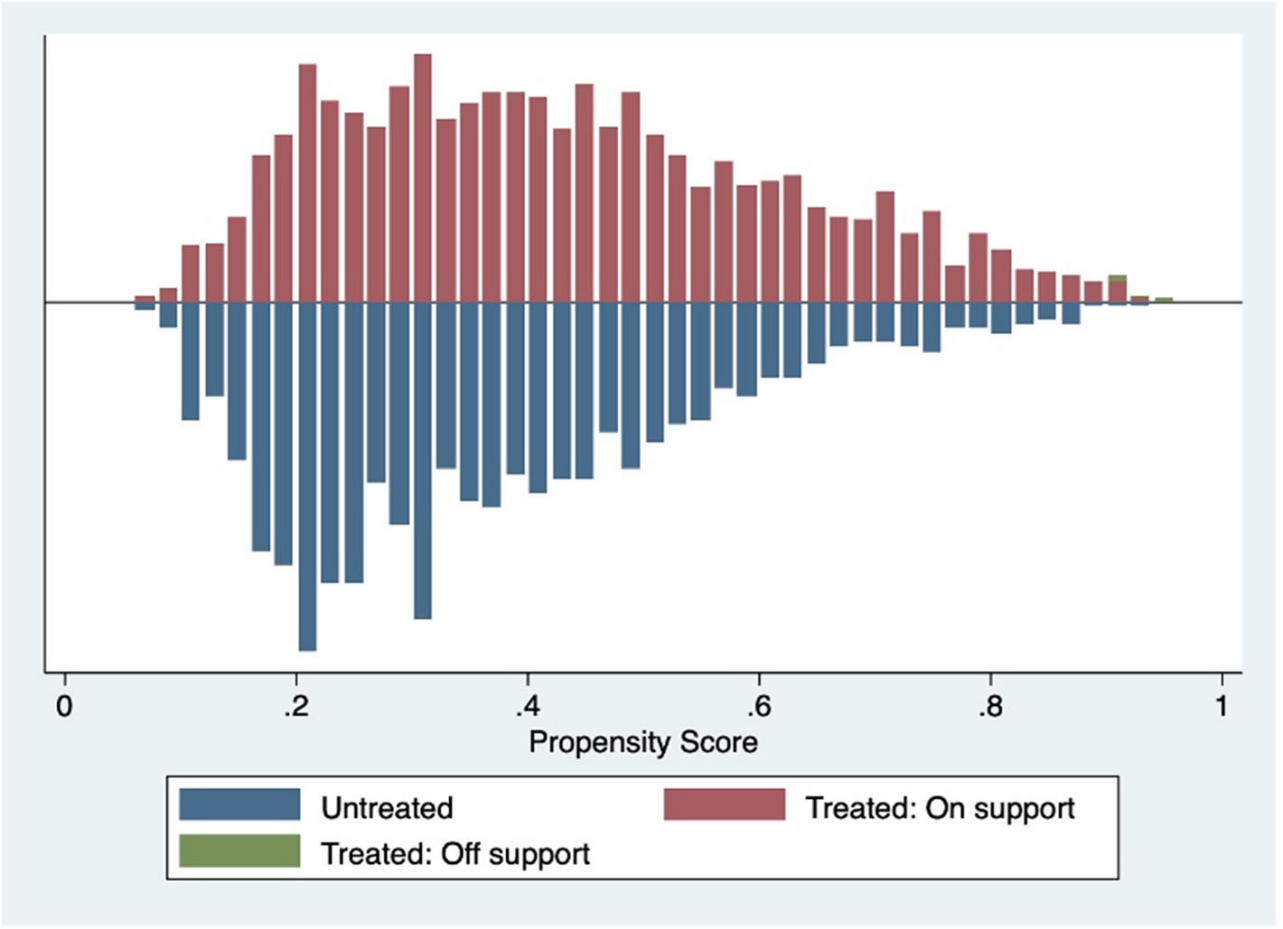
Distribution of propensity score matching into the two groups: unexposed (not transitioning, treated) and exposed group (transitioning, untreated)

Figure 1 shows a relatively homogeneous distribution of the PS between the two groups (blue bars for unexposed), thus guaranteeing the accuracy of matching methods; t-test for continuous variables and chi-square test for categorical ones suggest that the balancing property is met.

**Fig. 2:**
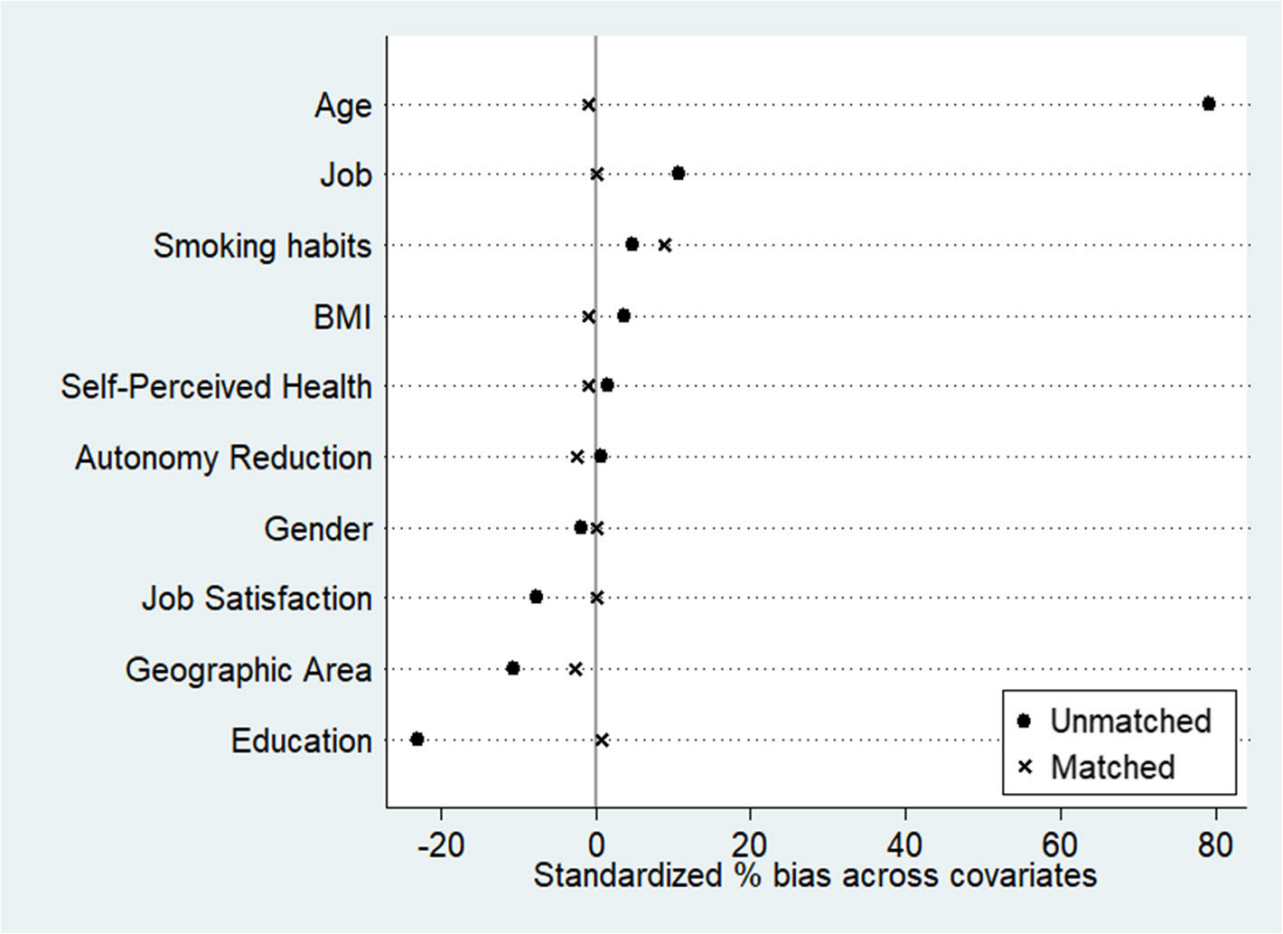
Percentage of bias reduction before and after propensity score matching

Figure 2 shows a significant bias correction from the unmatched to the matched sample. After PSM, the sample size was reduced from 5169 to 3270 subjects, with 1635 subjects in each group (retired and still at work, respectively). As shown in Table 1, no more significant statistical differences can be found between exposed and unexposed groups except tobacco smoking (*p* = 0.04).

Results from logistic regression models are reported in Table 2 and are referred to a weighted individual follow-up time mean of 2.11 years between retirement and Time1 (follow-up time was 1 year for 20.7% of the subjects, 2 years for 47.3% of them and 3 years for 32.0% of the total). Transitioning to retirement was associated with a 26% increase in the probability of reporting improved health status (RRR 1.26, 95% CI 1.02–1.58). Subjects who retired had a 36% greater probability of practising sport activities than subjects who did not exit the workforce (RRR 1.36, 95% CI 1.12–1.64). The risk of being overweight or obese did not change between exposed and non-exposed subjects (RRR 0.96, 95% CI 0.81–1.15). Subjects who transitioned to retirement reported a greater probability of smoking, although the difference was not statistically significant (RRR 1.18, 95% CI 0.94–1.46). Retired subjects also reported a RRR of 1.62 (95% CI 1.09–2.40) for physical functioning reduction after retirement, as compared to non-retired ones.

**Table 2:** Relative-risk ratios and 95% confidence interval from logistic regression for the association between status and outcomes

Outcomes	Exposed^**a**^ n. (%)	Unexposed n. (%)	RRR	95% CI	***p***-value
***Health status change***					
*No change*	773 (47.3%)	844 (51.6%)	1.00		
*Worsening*	465 (28.4%)	448 (27.4%)	1.13	0.92–1.40	0.25
*Improvement*	397 (24.3%)	343 (21.0%)	1.26	1.02–1.58	0.04*
***BMI***					
*Underweight-normal (< 25)*	762 (46.6%)	747 (45.7%)	1.00		
*Overweight-obese (≥25)*	873 (53.4%)	888 (54.3%)	0.96	0.81–1.15	0.68
***Current smoker***					
*No*	1284 (78.8%)	1317 (81.4%)	1.00		
*Yes*	345 (21.2%)	301 (18.6%)	1.18	0.94–1.46	0.15
***Sport habit***					
*No*	1022 (62.7%)	1124 (69.5%)	1.00		
*Yes*	608 (37.3%)	493 (30.5%)	1.36	1.12–1.64	< 0.01*
***Physical functioning change***					
*No change*	1464 (89.5%)	1501 (91.8%)	1.00		
*Worsening*	91 (5.6%)	58 (3.5%)	1.62	1.09–2.40	0.02*
*Improvement*	80 (4.9%)	76 (4.7%)	1.08	0.73–1.61	0.71

^a^transitioned to retirement

*RRR* Relative-risk ratio, *BMI* Body mass index

## Discussion

Analysing data from a large cohort of Italian adults, we observed the transition to retirement to be associated with a greater probability of physical activity and perceived health status improvement, although also with worse physical functioning.

With regard to behavioural risk factors, our findings concerning physical activity are supported by studies that have found that retirement has significant positive effects on voluntary physical activity [22–25]. Indeed, retirement results in a substantial reduction in hours worked and thus might provide retirees with the opportunity to devote more time to physical activity; increased leisure-time physical activity consequent to retirement is identified as one of the major positive health-related changes in behaviour related to retirement [6, 25, 26]. However, differences are reported by type of physical activity: evidence suggests that transition to retirement is associated with exercise and leisure time, but not with total physical activity [4], for which no clear pattern seems to emerge [2].

Besides, such association is influenced by socioeconomic status (SES), which may act as an effect modifier, with lower SES decreasing the strength of the association [2, 4]. Among retirees of low SES, the decline in occupational activity after retirement is not replaced by an increase in other physical activity domains, while increased leisure-time physical activity is observed among people of high SES [4, 22, 26–28]. Our model accounted for SES with multidimensional proxies built as categorical variables, such as educational level and job type [29].

Systematic reviews on the topic [2, 3] reported various patterns of changes in sedentary time and physical activity across retirement, identified using different study’s settings and methodologies (e.g., total sitting time vs specific leisure sedentary activities), reporting either a decline or an increase in duration, prevalence or frequency of physical activity. What is more, participation in physical activity varies by type of transition out of full-time employment [30, 31]: for instance, the case of disability retirement is quite a strong one [6]. On the one hand, a decline in physical activity is observed among people exiting from paid work due to a disability [30]. On the other hand, midlife employees who increase their physical activity have a lower risk of subsequent disability retirement than those persistently low-active and vice versa [6], suggesting the importance of promoting vigorous physical activity among adult employees.

Moreover, recent studies suggested that the observed increase in leisure-time physical activity after retirement is temporary and diminishes over time. The transient positive effect, which is greater among those retiring at older ages, from higher status occupation and with fewer chronic diseases, may be short-termed and not persist in post-retirement years [23, 24]. Our results refer to a follow-up from transition to retirement of 3 years, maximum, so they cannot be exploited to evaluate effects over a longer term.

Secondly and directly linked to physical activity and possibly acting as mediators, smoking habit and a high BMI can cause a decline in the levels of physical health [32, 33] and should be considered in examining retirement-related changes in physical activity [6].

In detail, retirement could affect tobacco consumption, albeit results are still inconclusive as Xue et al. [2] systematically observed a decrease or no effect on smoking habit.

Studies report contrasting evidence concerning BMI: BMI seemed to increase in lower socioeconomic groups or have no change in higher ones [34], but no clear pattern emerged. Previous work types may affect the physical practice, also through BMI. The loss of work-related physical activity from physically demanding jobs is not compensated by leisure-time physical activity, which would require substantial lifestyle adaptations, with a consequent increase in BMI if eating patterns are not changed. Obesity and overweight moderate the benefit of retirement on health and, with changes in BMI, all subjects are likely to experience improvements in self-rated and mental health [5].

Concerning self-rated health outcomes, on the one hand, a positive effect of retirement on self-rated health status has also been described by other European studies based on prospective data, such as the French GAZEL cohort [35] and the Whitehall II study, where mental health functioning improved in retirees from high employment occupation [7]. Our findings are generally consistent with those reporting that retirement appears beneficial for mental [36, 37] and perceived health [35]. Self-rated health was proved to be associated with mortality and is a valid measure of wellbeing and morbidity [38]. The reasons for the increased benefit may be that the burden of perceived health problems is substantially relieved by retiring, when people are no longer exposed to physically or mentally stressful conditions and can spend more time engaged in healthy activities, such as physical practice. As far as we are aware, our findings about perceived health status after retirement are among the first ones based on an Italian nationwide cohort of individuals who experienced the transition to retirement in the last 10 years. A fair generalisation might be proposed to other settings with generous health and social security systems in developed countries, since we accounted for the main possible confounders with PS.

On the other hand, even though literature findings have not produced conclusive evidence regarding physical functioning after retirement, with some studies suggesting functional benefits to retirement living [12], physical function was observed as declining with no significant difference between still working and retired subjects [7]. The Health and Retirement Study data corroborate a greater increase in physical functioning difficulties during retirement than in full-time work, accounting for chronic diseases and lifestyle-related risks [8]. Besides, the physical functioning reduction can moderate the effect between former work-related behaviours and physical activity during retirement. Our results appraise a significant change in physical functioning reduction towards a worsening outcome after retirement. Nevertheless, we acknowledged that both before and after PSM, the sample of individuals who reported a partial or total limitation is scarce, and the confidence interval of RRR is quite broad.

Our work has both strengths and limitations. Within the study’s strengths, first of all, PLUS offers an extensive national database in terms of data, stratification and representativeness of the Italian working population aged 50 years or more. To the best of our knowledge, at the national level, this is one of the very few studies with a longitudinal design performed in Italy on the topic and the first analysis to exploit these data in a public health perspective. Another work’s related strength is that the information derived from the same periodically conducted survey through rigorous procedures that include reliable CATIs. Secondly, a specific strength is the final study design: starting from cross-sectional waves, we reconstructed a longitudinal cohort via the individual-level data linkage, allowing us to follow the same subjects through different panel waves in all the years they were included. Finally, this study used a PSM approach to address the lack of randomness in the exposure (i.e., retirement) and simulating an experimental design. Even if this method may still have some limits, PSM increases the level of evidence of a study and, in turn, increases the strength and generalisability of its results [39].

Our study has some limitations. Firstly, we relied on self-reported health measures and health proxies. Secondly, the outcomes we derived from the survey are mostly not quantitative measures but derived from validated questions, such as the self-reported health assessment of the World Health Survey [18]. Thirdly, the study design did not cover an extensive follow-up period but focused on the differences reported in two consecutive interviews, before and after retirement. Finally, since the statutory pension age is predictable, workers may adjust their health-related behaviours before retirement; hence the potential effect of retirement on health and lifestyle behaviours may not completely coincide with the timing of withdrawal from employment.

Transition to retirement is a major life event, followed by changes at social, psychological, and physical levels that profoundly affect health. The circumstances of the transition can influence health behaviours [9], which are fundamental to maintain an acceptable level of health. Retirement triggers a complex set of adjustments and leads retirees’ priorities and way of life to change as well [3], with conflicting results presented in literature and possibly involving a wide range of domains [2–4, 9, 10].

Adopting new healthy lifestyles is not easy at an older age. Thus, health promotion at this stage is a public health priority; the transition to retirement has an independent effect in itself and, as such, could be identified as a target point for prevention [15]. As life-course transitions tend to bring along lifestyle changes, synchronising them with public health interventions might be a successful approach [40]. Although finding occasion for promoting the initiation and maintenance of healthy lifestyles is needed across the life course to prevent short- and long-term risk of unhealthy changes, this study supports previous evidence that the process of retirement is a window of opportunities for primary prevention interventions which could be effectively directed towards transitioning subjects [15].

Physical﻿ activity is a critical component of healthy ageing [41] and a key to preserve and improve health at older ages. Suggesting that the transition to retirement is associated with an increase in a moderate level of physical activity probably linked to people’s free time, our study focuses on the need for intervention studies to test whether retirement offers an optimal and favourable moment for boosting the natural increase in physical activity [42].

Overall, gaps in knowledge persist. More extended longitudinal studies might help disentangle the different elements that mediate the effects of retirement on risk factors and health outcomes and analyse the temporal evolution, possibly differentiating contextual and individual characteristics. This effort would contribute to the implementation of prevention measures to promote healthy ageing. Finally, the role of health inequalities must be researched in depth to design public health policies targeting disadvantaged groups.

## Conclusion

Retirement effects on health and health-related behaviours are crucial for the future sustainability of healthcare and pension systems in most Western countries. The varied and long-term impacting consequences of retirement show that an economic perspective might be narrow-minded to guide future reforms [43]. The public health implications of retirement might be considered in a multidimensional and multiprofessional way to address the demographic and epidemiologic transition.

Even if there may be some functional benefits to retirement living, new prevention strategies to encourage healthy lifestyles in later life and maintain daily physical activity in the long term are needed across all SESs. Future research should focus on the determinants and pathways of behavioural changes after retirement to inform this development. Linking multiple and future further waves of national surveys may offer great insight to direct healthy ageing policies in Italy and Europe, possibly allowing a more extensive follow-up record linkage.

## Supplementary Information

**Additional file 1.**

## Data Availability

The datasets supporting the conclusions of this study are publicly available from INAPP (https://inapp.org/it/dati/plus) upon request.

## Abbreviations

PLUS: Participation, Labour, Unemployment Survey
INAPP: Italian National Institute for Public Politics Analysis
CATI: Computer-Assisted Telephonic Interview
BMI: Body Mass Index
PSM: Propensity score matching
PS: Propensity score
RRR: Relative-risk ratios
SES: Socioeconomic status
