# Supplementary material for: Transition to retirement impact on health and lifestyle habits: analysis from a nationwide Italian cohort
Source: BMC Public Health. 2021 Sep 14;21:1670. doi: 10.1186/s12889-021-11670-3 (PMC8439097; doi:10.1186/s12889-021-11670-3)
Supplement: Supplementary file 1 — Additional file 1. [file 12889_2021_11670_MOESM1_ESM.docx]

**Supplementary Table S1: INAPP PLUS questionnaire’s items used in the analysis for exposure and outcome variables and covariates with categorisation details applied.**

| **Questions** | **Possible answers** |
| --- | --- |
| Which is your township of residence? | Area of residence was automatically derived from this answer: North-West (Liguria, Lombardia, Piemonte, Valle d'Aosta; North-East -Emilia-Romagna, Friuli-Venezia Giulia, Trentino-Alto Adige, Veneto), Center (Lazio, Marche, Toscana ed Umbria), South and islands (Abruzzo, Basilicata, Calabria, Campania, Molise, Puglia, Sardegna, Sicilia). |
| Which is your prevalent status? | Employed (exposure) |
|  | Searching for a job |
|  | Retired from paid job |
|  | Not working |
|  | Student |
| How old are you? | years |
| Sex | Male |
|  | Female |
| Which is your highest education degree? | None (Low) |
|  | Elementary (Low) |
|  | Middle school (Low) |
|  | High school (Medium) |
|  | University graduation (High) |
|  | Post-graduate degree (High) |
| What is your job? | Legislators, entrepreneurs, high management (White collars) |
|  | Intellectual, scientific and high specialized profession (White collars) |
|  | Technical profession (White collars) |
|  | Manager (white collars) |
|  | Commercial and services qualified profession (White collars) |
|  | Artisans, qualified workers and farmers (Blue collars) |
|  | Plant manager, equipment worker, driver (Blue collars) |
|  | Unqualified profession (Blue collars) |
|  | Armed forces (Blue collars) |
| What is the overall satisfaction level about your job? | High |
|  | Medium-high |
|  | Medium-low |
|  | Low |
|  | I don’t know |
|  | Not applicable |
| In general, how would you rate your health today? | Very good (Excellent) |
|  | Good |
|  | Moderate (Satisfactory) |
|  | Bad (Poor-Bad) |
|  | Very bad (Poor-Bad) |
| Do you have…? | A partial or temporary reduction in physical functioning (Partial limitation) |
|  | A permanent reduction in physical functioning (Total limitation) |
|  | No reduction in physical functioning |
|  | Not answering |
| Do you usually practise sports? | Yes |
|  | No |
|  | Not answering |
| Do you usually smoke? | Yes |
|  | No |
|  | Not answering |
| What is your weight? | kg |
| What is your height? | cm |
